# Spin-Resolved Quantum Scars in Confined Spin-Coupled Two-Dimensional Electron Gas

**DOI:** 10.3390/nano11051258

**Published:** 2021-05-11

**Authors:** Michael Berger, Dominik Schulz, Jamal Berakdar

**Affiliations:** Institut für Physik, Martin-Luther-Universität Halle-Wittenberg, 06099 Halle, Germany; michael.kraus@physik.uni-halle.de (M.B.); dominik.schulze@physik.uni-halle.de (D.S.)

**Keywords:** quantum scars, spin-orbit coupling, quantum chaos, periodic orbits

## Abstract

Quantum scars refer to an enhanced localization of the probability density of states in the spectral region with a high energy level density. Scars are discussed for a number of confined pure and impurity-doped electronic systems. Here, we studied the role of spin on quantum scarring for a generic system, namely a semiconductor-heterostructure-based two-dimensional electron gas subjected to a confining potential, an external magnetic field, and a Rashba-type spin-orbit coupling. Calculating the high energy spectrum for each spin channel and corresponding states, as well as employing statistical methods known for the spinless case, we showed that spin-dependent scarring occurs in a spin-coupled electronic system. Scars can be spin mixed or spin polarized and may be detected via transport measurements or spin-polarized scanning tunneling spectroscopy.

## 1. Introduction

Wave localization by disorder is a ubiquitous phenomenon widely discussed for quantum [1] and for classical waves [2,3,4] and can be interpreted by scattering and interferences without involving many-body interactions. Correlated quantum systems may exhibit disorder-induced many-body localization involving states with a high energy level density [5,6,7,8,9,10,11,12,13,14], as well as correlation-induced localized states, called quantum scars [15]. Scars in wave functions, meaning enhanced localization of the probability density for states in the high energy level density part of the spectrum, were first discussed for non-interacting systems [16,17,18,19,20] and interpreted by analyzing the classical orbits of an electron or waves in various confinements such as chaotic billiards [16,17,18,19,20,21,22,23,24,25,26]; for a discussion, we refer to the book by Heller [27]. Scars occur for confined systems with and without disorder and were experimentally investigated for various systems, including microwave resonators [28,29]. Conventionally, disorder is introduced by adding localized scalar perturbations to the potential. Signatures or traces of scars in open nanoscale systems can be picked up in transport measurements [30,31,32,33,34,35] in the linear response regime, in which case the measured signal is proportional to the local density of states. Scars might be useful as a transport channel for hot electron tunneling since strong wave packet recurrence along the trajectory is expected [25]. The quantum nature of the localized state is also interesting for quantum information studies. The focus of this work was on the role of spin in spin-charge coupled systems, which has not received attention yet in connection with scarring, but may potentially be interesting for spintronics; for example, if scarring would lead to localized, spin-polarized states, which could influence the spin-polarized conductance. A potentially spin-polarized scar can be detected via spin-polarized scanning tunneling spectroscopy [36,37]. As a case study, we considered a semiconductor-based two-dimensional electron gas (2DEG) in the confinement potentials detailed below and subject to an external magnetic field and uncorrelated scalar impurities. Due to the symmetry breaking at the interface of the two semiconductors where the 2DEG is formed, a Rashba-type spin-orbit coupling (SOC) is operational. We calculated the single particle states in the high energy level density regime and analyzed the spectral properties, as well as spin-dependent perturbation-induced scarring, which was indeed found to be spin-channel selective.

In Section 2, we introduce the theoretical models and numerical tools for the calculation of the spin-dependent scars that we analyzed and discuss various settings in Section 3, followed by a summary in Section 4.

## 2. Theoretical Model

As a confining potential for the 2DEG, we considered a square surface with different boundary conditions. The magnetic field B is perpendicular to the 2DEG film. The Hamiltonian for the single-particle states subjected to the magnetic field and the impurities potentials $V_{n}$ read (atomic units (a. u.) $\hbar =m_{e}=e=1$ are used) [38]:

(1) $$\begin{array}{cc} H & =\frac{\Pi^{2}}{2m^{*}}+\alpha {\left(\sigma \times \Pi \right)}_{z}+\sum_{n=1}^{N_{\mathrm{Imp}}}M_{n}V_{n}\left(x,y\right)+\frac{1}{2}g\mu_{B}B\sigma_{z}, \end{array}$$

where $m^{*}$ is the effective electron mass, σ are the standard Pauli-matrices, and Π=p−eA is the kinetic momentum, with B=∇×A. The Rashba spin-orbit coupling strength is α. $\mu_{B}$ is the Bohr magneton, and *g* is the Landé factor. The parameter α may be varied by applying a gate voltage to the nano-structure. Electrons in 2DEG are constrained to move in the xy plane. For the vector potential, we used the asymmetric gauge $A=B{\left(-y,0,0\right)}^{T}$ to correctly implement the periodicity. The impurities may have individual strengths $M_{n}$. The spatial distribution used in the calculations below has the Gaussian form:

(2) $$\begin{array}{cc} V_{n}\left(x,y\right) & =\exp\left\{-\frac{{\left(r-r_{n}\right)}^{2}}{2\sigma_{n}^{2}}\right\}, \end{array}$$

where the impurities are distributed randomly around the positions $r_{n}$ and have a full width at half maximum (FWHM) of $\omega_{n}=\sigma_{n}\sqrt{2\log2}$. For simplicity, all impurities have the same strength and FWHM (and thus, we dropped the index *n* for the strength and FWHM). Two different boundary conditions were considered: Dirichlet-type and one-side periodic. The numerical results discussed in the following sections are for the case: $m^{*}=1$, square- or rectangular-shaped confinements with the extension 100a.u. by 100a.u. or 50a.u. by 100a.u. The Rashba SOC parameter α ranges from 25 to 200meV. The magnetic field strength is 0.5 to 2T, and $N_{\mathrm{Imp}}=10$ impurities were assumed with a strength ranging from 0 to 0.2a.u. and a width varying between 0.05a.u. and 0.3a.u. The values used throughout this paper are exemplary. For example, the eigenvalues of the states shown in this paper can be scaled down by increasing the system size and all other parameters accordingly. The calculations were performed using a finite difference scheme with a third-order discretization with a grid of 501 points in each dimension and solving the eigenvalue problem with the SLEPc-library [39].

## 3. Scars

### 3.1. Scar Detection

To identify and quantify the scars and the spectral distributions, statistical parameters were introduced [23,40]: the absolute fourth power of the wavefunction, also known as the inverse participation ratio (IPR) [41], is given by:

(3) $$\begin{array}{cc} I_{\Psi } & =\frac{\int {\left|\Psi (r)\right|}^{4}dV}{\int {\left|\Psi (r)\right|}^{2}dV}. \end{array}$$

For an even distribution, the IPR is $I_{\mathrm{even}}=1/V$ (*V* is the system’s volume), and localized states have a higher value depending on the degree of localization. Strongly scarred states have a significantly increased IPR, but our calculations revealed that states with the highest IPR were just randomly localized. Thus, it was beneficial to select the scarred states by plotting them. Hence, the IPR was not shown in this paper.

Another method is to analyze the energy spectrum based on the distances of consecutive energy levels. The spectrum of the nearest neighbor level spacings (NNLS) has been extensively studied for rectangular systems [21,22] or microwave resonators [28,29]. Reference [42] discussed the NNLS distribution P(s) as a function of the level spacing *s* normalized by the mean spacing $\bar{s}$:

(4) $$\begin{array}{c} \begin{array}{cc} P_{\mathrm{BR}}\left(s\right) & =e^{-qs}\left[q^{2}\mathrm{erfc}\;\left(\frac{\sqrt{\pi }}{2}\bar{q}s\right)+\left(2\bar{q}q+\frac{\pi }{2}{\bar{q}}^{3}s\right)\exp\;\left(-\frac{\pi }{4}{\bar{q}}^{2}s^{2}\right)\right],\\ \bar{q} & =1-q. \end{array} \end{array}$$

$P_{q}\left(s\right)$ transforms from a Poissonian ($q_{\mathrm{BR}}=0$) to a Wigner-type distribution ($q_{\mathrm{BR}}=1$), depending on the mixing parameter $q_{\mathrm{BR}}$. The distributions coexist for the regular and chaotic underlying classical dynamics of the system. Alternatively, the Brody distribution as an interpolating function between the integrable and non-integrable regimes of a quantum system can be used to characterize our systems [43]. For $q_{B}=0$, it equals a Poissonian distribution, and for $q_{B}=1$, a Wigner distribution,

(5) $$\begin{array}{cc} P_{\mathrm{Brod}.} & =\left(q+1\right)a_{q}s^{q}e^{-a_{q}s^{q+1}}, \end{array}$$

where $a_{q}=\Gamma {\left(\frac{q+2}{q+1}\right)}^{q+1}$ and Γ is the Gamma function. In the following, the mixing parameters are denoted as $q_{\mathrm{BR}}$ (Berry–Robnik mixing) and $q_{B}$ (Brody mixing). Numerically, the NNLS were calculated from the ordered energy eigenvalues. The spectrum was divided into two subspectra depending on the *z*-component of the spin of the respective eigenstate. Then, these (spin-polarized) spacings were normalized to their mean value $\bar{s}$.

A further statistical measure addressing the spectral rigidity $\Delta_{3}^{\mathrm{system}}\left(L\right)$ of the energy spectrum with:

(6) Δ3system(L)=Δ3Poisson(QL)+Δ3GOE1−QLM33,

is basically an interpolation between a Poisson distribution and a Gaussian orthogonal ensemble with *Q* being the interpolating factor with the same range as $q_{\mathrm{BR}}$. Numerically, for each data point at $L_{i}$, Equation (6) was solved. Then, the mean value was calculated from all ${q_{i}}^{\prime }s$. A further approach used in Reference [23] is to analyze the spectral fluctuations as a time series. The spectral rigidity $\Delta_{3}\left(L\right)$ is then proportional to $L^{2\alpha }$ [44]. Therefore, the measured rigidity is fitted to the function:

(7) $$\begin{array}{cc} f_{\alpha }\left(L\right) & =\beta L^{2\alpha }+\Delta_{3}^{0}. \end{array}$$

Having obtained the spectrum numerically, we determined the (spin-dependent) mixing parameter entering Equations (4) and (5) for each spin-channel separately, which is a reasonable approach considering that the splitting of the otherwise degenerate states is much smaller than other energy level spacings [45]. All the statistical measures presented are suited to classify the quantum systems; however, we found the Berry–Robnik mixing (Equation (4)) to be the most suitable one.

### 3.2. Level Statistics

**Figure 1:**
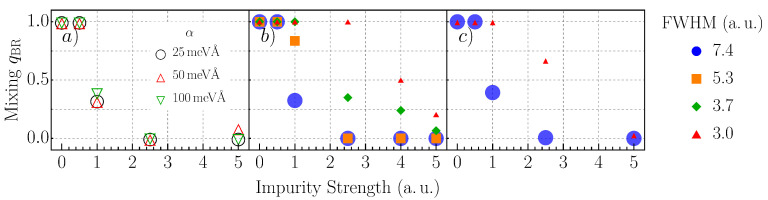
The mixing parameter $q_{\mathrm{BR}}$ as it enters Equation (4). We consider a 2DEG confined to a square with infinite boundaries with a size of 100×100 a.u., which contains ten randomly distributed Gaussian impurities with varying strengths and FWHM, as indicated. A magnetic field with a strength of B=500mT is applied transverse to the 2DEG. Panel (**a**) shows the dependence on the strength α of the Rashba SOC for a fixed FWHM=7.4a.u. of the Gaussian impurities. Panels (**b**,**c**) show the dependence of $q_{\mathrm{BR}}$ on the impurities’ strength and FWHM for α=25 in (**b**) and meVÅ and α=100meVÅ in (**c**).

For squares and rectangle confinement potentials, the nature of the classical trajectories for a spinless particle is established. Our focus was, therefore, on spin-dependent scarring and the suitable conditions for its occurrence. For B=500mT, one-thousand eigenstates were calculated for each system, meaning about 500 energies for each spin channel were used to infer the statistical parameters from . For this analysis, we found that this number of energy levels already gave high confidence estimates. This problem was discussed in Reference [21]. The results are shown in Figure 1. In general, if the impurities are too weak, they do not disturb the system strong enough to cause noticeable changes in the NNLS distribution, as shown in the leftmost plot of Figure 1.

The threshold for the impurity potential strength depends on the FWHM. In the center plot of Figure 1, the change of the mixing parameter $q_{\mathrm{BR}}$ entering Equation (4) is shown as a function of the impurity potential strength and extension. The point where the NNLS distribution is no longer a pure Poissonian (q=1) changes from M≃0.75 for ω=7.4a.u. over M≃1.5 (ω=3.7a.u.) to M≃3.25 for an FWHM of 3.0a.u.

For extended impurity potentials (large FWHM), the potentials act as a smooth background for the electrons. This holds true as long as the impurities are sufficiently weak. With an increasing amplitude, the eigenstates are strongly perturbed, and the degeneracies are lifted, even for states with energies well below the perturbation potential. In contrast, the impact of abruptly raising impurity potentials is clearly observable for a smaller impurity potential strength (see Figure 1). The role of SOC on the impurities scattering is marginal. We found scarring is possible even for a rather large SOC parameter α.

### 3.3. Scars

**Figure 2:**
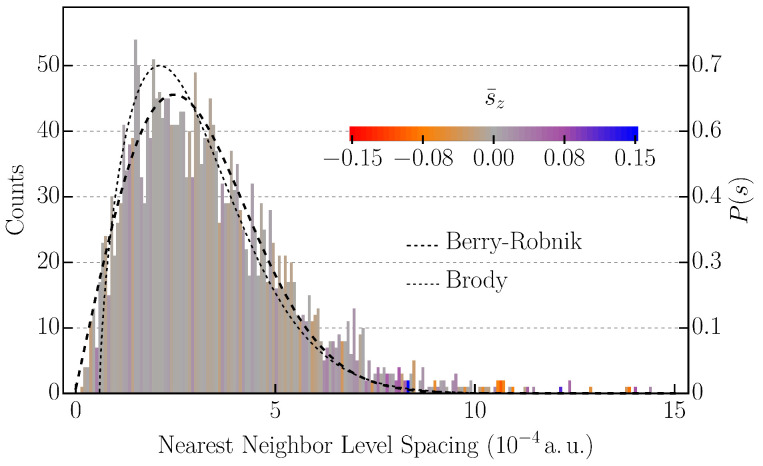
Nearest neighbor level spacing histogram for a square with a size of 100×100 a. u. with periodic boundary conditions in the *x*-direction, α=20meVÅ, B=1T, with ten impurities with a strength of 0.18a.u. and FWHM = 8a.u. In total, three-thousand and six energies are distributed into 200 bins of width $7.2\times 10^{-6}\;a.\;u.$ The mixing parameter $q_{\mathrm{BR}}$ (Equation (4)) is equal to 0.134, and the Brody parameter (Equation (5)) $q_{B}$ equals 0.637. The color code shows the mean of all $s_{z}$ values of all states belonging to the bin. The (mean) spin-polarization is higher when the states have small or very large energy spacing.

For a rectangular system with Dirichlet boundary conditions, the periodic orbits with or without a magnetic field have been well analyzed, for instance in [46]. It is well known that scarring occurs in these systems. We considered a system with a length of 100a.u., containing 10 impurities with an impurity potential strength of (M=5eV (or 0.18a.u.), ω=8a.u.) and a magnetic field in the *z*-direction with a strength of 0.1T, and α=25meV. Comparing with Figure 1, scarring was expected, but the wavefunction would not be such that most of the probability density is strongly localized around the classical path. For better statistics, three-thousand and six states (1503 in each spin channel) were calculated. The NNLS distribution was dominated by a peak near s=0, since the splitting of spin-up and spin-down states was weak due to the weakness of SOC strength, as well as the weak magnetic field. A fit of Equation (4) yielded the value q=0.077 for the spin-polarized spectra. The energy ranged from ∼0 to 1a.u. Stronger SOC or altering the boundary conditions can remove the peak at s=0, which is the case in Figure 2.

**Figure 3:**
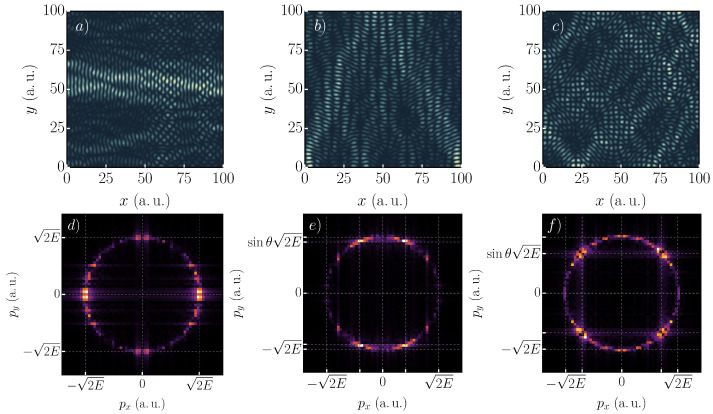
Three scarred eigenstates of a square with a size of 100×100 a.u., α=25meVÅ, B=100mT, ten impurities of strength M=0.18a.u. and FWHM=2.6a.u., with Dirichlet boundary conditions. Lighter colors represent higher density, while the background color (blue for **a**–**c** and black for **d**–**f**) means zero density. In each panel (**a**–**c**), at least one prominent path is visible. The 2834th (**a**), 2887th (**b**), and 2949th (**c**) states are shown with energies of 0.91, 0.93, and 0.95a.u. In Panel (**c**), two paths parallel to the diagonals are visible. The wave functions in the momentum space for the states are shown in Panels (**d**–**f**).

Examples of possible scars are shown in Figure 3. The top row shows the probability density of the 2834th (a), 2887th (b), and 2949th (c) state with energies of 0.91, 0.93, and 0.95a.u. One can identify classical paths for these probability densities. In Figure 3a, a straight line at y=55a.u. is visible. Figure 3b shows a path in the *y*-direction with one reflection at x=50a.u. Inspecting Figure 3c, the classical path does not seem to be fully connected. In fact, there are two independent closed paths. The paths are rotated by an angle of $90^{\circ }$ with respect to each other. To unravel the scars, we analyzed the Fourier transforms of each state, as depicted in the bottom row; see Figure 3d–f. The classical momenta expected to be dominant for scarred states are shown. Classically, all momenta should lie within a circle of radius $p_{\max}=\sqrt{2m^{*}E}$, (with $m^{*}=1$). The coordinates are scaled according to the energy of the Fourier-transformed state.

The first state revealed a very prominent contribution of momenta with $p_{x}=p_{\max}$ and $p_{y}=0$. This set of momenta belongs to the vertical path of Figure 3a. Another perpendicular path can be inferred from the Fourier transform (Figure 3f) at x=60a.u., but with low contrast with respect to the noise in the coordinate representation. This situation is generally valid for the scarred states in the considered case. The second state showed the expected momenta on the $p_{\max}$ circle at a reflection angle $\theta =60^{\circ }$. The last state had its most contributing momenta at reflection angles of $45^{\circ }$. From the Fourier-transformed probability density, the decoupled paths could not be distinguished.

**Figure 4:**
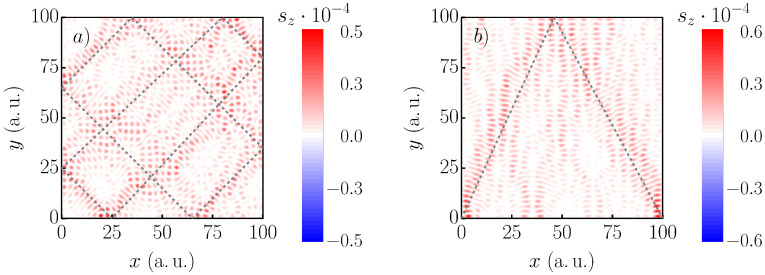
Spatially resolved $s_{z}$ component of the states 2887 and 2949 of a square with a size of 100×100 a.u., α=25meVÅ, B=100mT. Ten impurities of strength M=0.18a.u. and FWHM=2.6a.u. are randomly positioned inside the square. The classical trajectories are indicated by dashed lines. The trajectory in Panel (**a**) is open, while in (**b**), two closed paths are shown. Both states are mostly in the spin-up state, which corresponds to $\langle s_{z}\rangle ≃1$. Note the different scale of the color bars. The oscillations of the lower state are ≈0.05a.u. stronger.

Inspecting the spin-resolved probability density (Figure 4), we found full spin polarization along the classical paths. Due to the weak SOC strength α, spontaneous spin flip processes along the classical trajectory were unlikely. However, the scar in Figure 4b is spin-up dominated, but the corners of the rectangular paths are in the spin-down state. Both the spin-up and spin-down density are of the same magnitude.

Considering periodic boundary conditions with only one periodic side, the system resembled a 2DEG on the surface of a cylinder with length L=100a.u. and radius R=16a.u. This changed the shortest periodic orbits and their distributions along the symmetry axes. The shortest periodic orbits were straight lines in either direction, in the *y*-direction with two reflections and in the *x*-direction with zero reflections. Thus, most straight line scars were expected to move around the cylinder. Ten impurities with the same parameters as before were placed on the surface; α=0.2meVÅ; and a transversal magnetic field of B=1T was applied. The maximum energy was 0.8a.u. for the 2502nd eigenstate. Calculating the statistical mixing parameters of Equation (4) yielded a value of q=0.134 for each spin-polarized spectrum (containing 1263 eigenvalues). This parameter is a very sensitive indicator. The spectral rigidity (Equation (6)) showed clearly that the considered system was neither Poissonian nor fully chaotic as may have been inferred from a fit to the Berry–Robnik distribution. The NNLS histogram of the periodic system is shown in Figure 2. There, we chose 200 bins with a width of $7.2\times 10^{-6}\;a.\;u.$ Note that using 150 or 250 bins did not change the distribution qualitatively, and with that, the mixing parameter seemed to be mostly independent of the choice of bins. Additionally, the colors show the mean value ${\bar{s}}_{z}^{k}=\sum_{j=1}^{N_{k}}s_{z}^{j}$ of all states contributing to the *k*’th bin, e.g., when two level spacings are in bin *k*, then four states contribute to the mean spin. Hence, ${\bar{s}}_{z}^{k}$ is a measure for the mean spin-polarization. From Figure 2, it can be concluded that the states that have very small or very large energy-spacing were more strongly spin polarized.

**Figure 5:**
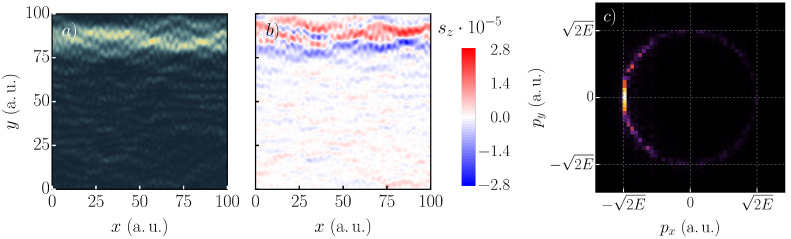
Probability density (**a**) of the 1821st state ($s_{z}=-1$), spin-polarized density (**b**), and the Fourier-transformed spin density (**c**) for a square system of lengths 100a.u. with periodic boundary conditions in the *x*-direction, α=20meVÅ, B=1T with ten impurities with a strength of 0.18a.u. and FWHM = 8a.u. The energy is E=0.59a.u. The momentum distribution indicates that the particle moves along the −x-direction. The path at y=85a.u. consists of the spin-up (red) and spin-down (blue) channels, which are split.

Figure 5 shows the probability density of the 1821st eigenstate with the rightmost panel indicating the corresponding momentum distribution. From the symmetry, one expects at most two prominent contributions $\pm p_{x}$, which is characteristic for a particle moving from the left (right) to the right (left) on a straight line ($p_{y}=0$). This assumption was confirmed by the momentum distribution. Figure 5b shows the spin-polarized probability density $s_{z}\left(r\right)=|\Psi_{1821}^{↑}{\left(r\right)|}^{2}-{\left|\Psi_{1821}^{↓}\left(r\right)\right|}^{2}$. Interestingly, the scarred pattern remained intact. Moreover, the parts of the trajectory for the spin-up (red) and spin-down (blue) split. The results suggested that scarring occurs for each spin channel individually, causing the spin-polarized probability density to be scarred in the presence of the SOC and magnetic fields.

### 3.4. Local Density of States

Experimentally, the local density of states (LDOS) can be measured by microscopy or by pump-probe experiments. It can be calculated using the eigenstates $\Psi_{i}$ and a function f(ε) denoting a distribution dependent on the energy. Here, the spectrum consisted of discrete values $\varepsilon_{n}$, the eigenvalue of the *n*’th state. Hence, one needs to sum all states with their weight respectively, and this leads to the following equations:

(8) $$\begin{array}{cc} \rho (r) & =\sum_{n}{\left|\Psi_{n}\left(r\right)\right|}^{2}f\left(\varepsilon_{n}\right), \end{array}$$

(9) $$\begin{array}{cc} \rho {\left(r\right)}_{s_{z}} & =\sum_{n}\left(|\Psi_{n}^{↑}{\left(r\right)|}^{2}-{\left|\Psi_{n}^{↓}\left(r\right)\right|}^{2}\right)f\left(\varepsilon_{n}\right). \end{array}$$

The latter shows the spin-resolved LDOS (SR-LDOS), which can be measured using a magnetic tip. The distribution f(ε) can be the Fermi–Dirac-distribution, which will sum all states up to the fermi-energy, or one calculates local effects using Gaussian (or similar) distributions. With the latter form of distributions, the visibility of the scar can be determined if one probes a superposition of states. In reality, transport measurements cannot be performed at exact energies; hence, a single scarred state is hard to “select” in experiments. However, if these states “survive” over a larger energy range, the effects of the scarring could still play a role, even if the neighborhood of the scarred state is not scarred.

**Figure 6:**
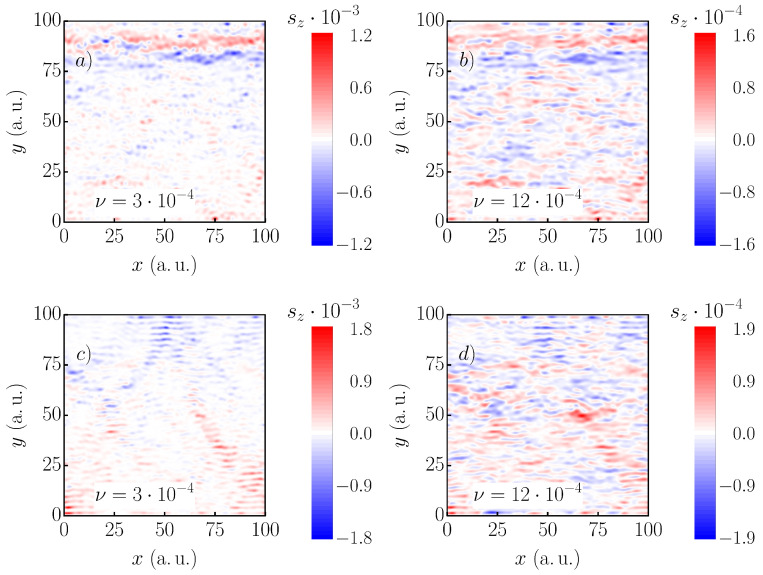
The local density of states for a square *x*-periodic system, lengths 100a.u. with α=20meVÅ, B=1T with ten impurities of strength M=0.18a.u., and FWHM = 8a.u. The density of the 1821st (**a**,**b**) and 2424th (**c**,**d**) are shown. Panels (**a**,**c**) were calculated using $\nu =3\times 10^{-4}$, (**b**,**d**) using $\nu =12\times 10^{-4}\;a.\;u.$, which corresponds to 16 and 72 averaged states, respectively. The spin-polarization is decreased by one order of magnitude when the number of summed states is increased.

For these calculations, a Gaussian distribution function was chosen, reading:

(10) $$\begin{array}{cc} f(\varepsilon ) & =\frac{1}{\sqrt{2\pi }\nu }\exp\left\{-\frac{{\left(\varepsilon -\varepsilon_{k}\right)}^{2}}{2\nu^{2}}\right\}, \end{array}$$

where ν defines the number of states contributing to the LDOS and $\varepsilon_{k}$ the center energy. In Figure 6, the SR-LDOS for the 1821st (top) and 2424th state (bottom) are shown. As has been shown before, both states were scarred, while neighboring states were not. When 16 states were considered ($\nu =3\times 10^{-4}$) for the calculations, the paths were still visible in both cases. However, when the classical path of the scar became more complex, the structure vanished faster when averaging states. While the simpler straight path in Figure 6 is still noticeable when $\nu =12\times 10^{-4}$, almost no structure could be determined for the other state. The oscillations of the non-scarred states quickly superimposed the complex scar-structure. Still, this showed that scarring was stable against “impure” measurements. Further, the spin-polarization increased by a factor of 100 with respect to the single-state scar (see Figure 5b). This behavior is very interesting for future applications, for example in spintronic devices.

## 4. Summary

We studied the scarring of quantum states in a two-dimensional electron gas confined to a predefined geometry and subjected to an external magnetic field and Rashba-type spin-orbit coupling. Using the statistical methods of References [23,40,42,43,45] and applying them to spin-dependent states and spin-resolved spectra, we identified scarring in spin-dependent non-interacting electron systems. The analysis evidenced the existence of spin-mixed and spin-polarized scars, which could be of use in spin transport in nanoscale (spintronic) devices. Further, the local density of states was still scarred when calculated in the neighborhood of scarred states. Spin-resolved calculations even showed a huge increase in the average spin-polarization. However, the influence of other types of spin-orbit coupling such as Dresselhaus-SOC is still to be investigated.

## Data Availability

All data in this work are available from the authors upon reasonable request.
